# A Semi-Supervised Graph Convolutional Network for Early Prediction of Motor Abnormalities in Very Preterm Infants

**DOI:** 10.3390/diagnostics13081508

**Published:** 2023-04-21

**Authors:** Hailong Li, Zhiyuan Li, Kevin Du, Yu Zhu, Nehal A. Parikh, Lili He

**Affiliations:** 1 Imaging Research Center, Department of Radiology, Cincinnati Children’s Hospital Medical Center, Cincinnati, OH 45229, USA; 2 Department of Radiology, University of Cincinnati College of Medicine, Cincinnati, OH 45229, USA; 3 Neurodevelopmental Disorders Prevention Center, Perinatal Institute, Cincinnati Children’s Hospital Medical Center, Cincinnati, OH 45229, USA; 4 Artificial Intelligence Imaging Research Center, Cincinnati Children’s Hospital Medical Center, Cincinnati, OH 45229, USA; 5 Department of Computer Science, University of Cincinnati, Cincinnati, OH 45221, USA; 6 Department of Pediatrics, University of Cincinnati College of Medicine, Cincinnati, OH 45229, USA

**Keywords:** preterm infant, machine learning, deep learning, graph convolutional network, diffusion MRI, T2-weighted MRI, motor abnormality, cerebral palsy, neurodevelopment, prognosis

## Abstract

Approximately 32–42% of very preterm infants develop minor motor abnormalities. Earlier diagnosis soon after birth is urgently needed because the first two years of life represent a critical window of opportunity for early neuroplasticity in infants. In this study, we developed a semi-supervised graph convolutional network (GCN) model that is able to simultaneously learn the neuroimaging features of subjects and consider the pairwise similarity between them. The semi-supervised GCN model also allows us to combine labeled data with additional unlabeled data to facilitate model training. We conducted our experiments on a multisite regional cohort of 224 preterm infants (119 labeled subjects and 105 unlabeled subjects) who were born at 32 weeks or earlier from the Cincinnati Infant Neurodevelopment Early Prediction Study. A weighted loss function was applied to mitigate the impact of an imbalanced positive:negative (~1:2) subject ratio in our cohort. With only labeled data, our GCN model achieved an accuracy of 66.4% and an AUC of 0.67 in the early prediction of motor abnormalities, outperforming prior supervised learning models. By taking advantage of additional unlabeled data, the GCN model had significantly better accuracy (68.0%, *p* = 0.016) and a higher AUC (0.69, *p* = 0.029). This pilot work suggests that the semi-supervised GCN model can be utilized to aid early prediction of neurodevelopmental deficits in preterm infants.

## 1. Introduction

Preterm birth poses a high risk of developing motor abnormalities compared to infants who are term born [1,2]. Very preterm infants (<32 weeks gestational age) are especially at increased risk [3]. Globally, an estimated 32–42% of very preterm infants develop minor motor abnormalities [4,5]. Typical motor abnormalities, such as cerebral palsy and delayed motor development, and poor fine motor skills, are the result of abnormal brain development or brain injury during the fetal or neonatal period [6,7,8,9]. However, major motor impairments typically cannot be accurately diagnosed until 1–2 years of age, and minor motor impairments take even longer to diagnose [6]. Earlier diagnosis soon after birth is urgently needed, as the first 2 years of life represents a critical window of opportunity for early neuroplasticity in preterm infants [7]. Accurate and early prediction of motor abnormalities enables us to target early interventions to the highest-risk infants during periods of optimal neuroplasticity to improve their quality of life.

Diffusion tensor imaging (DTI) is an advanced MRI technique used to detect how water molecules travel along the white matter tracts in the brain [10,11]. It allows a more complex investigation of brain white matter microstructure and macrostructure [3,12,13]. In recent years, several studies have used DTI-derived features to predict motor abnormalities in preterm infants [14,15,16,17,18]. Despite the promising results of machine learning, especially deep learning, in the early prediction of motor abnormalities in neonates, the previous studies solely focused on analyzing the features of individual subjects in isolation, neglecting potential pairwise interactions between subjects.

A graph provides a natural framework for capturing pairwise relationships between individuals [19,20,21]. In a graph, individuals can be represented as nodes and pairwise relationships can be represented as edges (binary or weighted). The recent advent of graph convolutional networks (GCNs) [22,23] has led to advanced disease classification applications that simultaneously consider features of individual subjects and relationships between subjects. For example, Parisot et al. [24] constructed sparse population graphs for the diagnosis of autism spectrum disorder (ASD) and Alzheimer’s disease (AD). In a more recent work, Jiang et al. [25] developed a hierarchical GCN to further improve performance in ASD and AD classification tasks. These promising results inspired us to explore the capabilities of GCN models in the task of predicting motor abnormalities in very preterm infants.

For node classification tasks, GCN models are actually trained in a semi-supervised learning manner, enabling us to arbitrarily integrate more data from subjects regardless of the availability of their labels [22]. Prior early motor abnormality prediction models were all supervised learning models that used labeled training data only [14,15,16,17]. Semi-supervised learning methods can produce considerable improvement in classification accuracy by using additional unlabeled data together with labeled data [26,27]. Thus, we set out to explore whether a semi-supervised GCN model is able to improve prediction of motor abnormalities by leveraging the available unlabeled data.

In this study, we developed a semi-supervised GCN model for predicting motor abnormalities at 2 years corrected age using DTI data obtained from very preterm infants at term-equivalent age. We conducted our work on a multisite regional cohort of preterm infants born at 32 weeks or earlier from the Cincinnati Infant Neurodevelopment Early Prediction Study (CINEPS). We sought to test our hypothesis of whether such a semi-supervised GCN model can improve prediction performance over peer supervised machine learning and deep learning models. We also hypothesized that the integration of labeled and unlabeled data will boost the performance of GCN models that used labeled DTI data alone.

## 2. Materials and Methods

### 2.1. Overview

**Figure 1:**
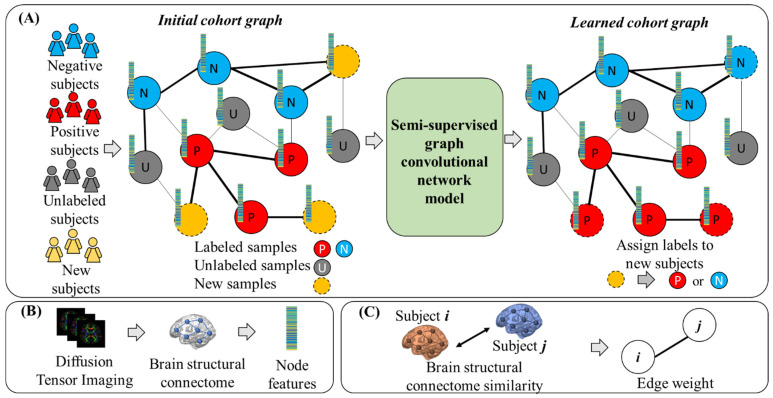
The proposed semi-supervised learning framework to predict motor impairment at 2 years corrected age using brain structural connectomes derived from DTI data acquired at term in very preterm infants. Given both labeled (blue and red) and unlabeled (gray) subjects ready for model learning, our task was to classify new subjects (yellow) into one of the label groups. (**A**) We constructed an *initial cohort graph*, including labeled subjects (positive (P) and negative (N)), unlabeled (U) subjects, and to-be-predicted new subjects. The semi-supervised GCN model learned the *initial cohort graph* and assigned labels (i.e., positive for high risk and negative for low risk of developing motor impairment) to new subjects in the *learned cohort graph*. (**B**) We used diffusion tensor imaging (DTI)-derived brain structural connectomes as node features and (**C**) the similarity between brain connectomes as edge weights between nodes.

An overview of our semi-supervised GCN framework for early risk prediction of motor impairment is illustrated in Figure 1. When this work commenced, only about half of the cohort had received 2-year motor assessment (labeled subjects), while the other half had not reached 2 years corrected age (unlabeled subjects). For subject (node) classification tasks, GCN models are trained in a semi-supervised learning manner, a strategy between supervised learning (with only labeled data) and unsupervised learning (with only unlabeled data). This allowed us to utilize the whole cohort regardless of their label availability. Given both labeled (blue and red) and unlabeled (gray) subjects ready for model learning, our main task was to classify new subjects (yellow) into one of the label groups. We first constructed an *initial cohort graph*, represented by a weighted graph $G\left(𝓋,E,\;W\right)$ (Figure 1A). In this graph, each node 𝓋 represents a subject associated with a set of node features, and the weighted edge E represents the similarity between two subjects associated with a weight W reflecting the similarity scale. This *initial cohort graph* includes labeled subjects (positive (P) and negative (N)), unlabeled subjects (U), and new to-be-classified subjects. We formulated our motor abnormalities prediction task as a graph node binary classification problem, where the semi-supervised GCN model learned the *initial cohort graph* and assigned labels (i.e., positive for high risk and negative for low risk of developing motor abnormalities) to subjects in the *learned cohort graph*. We obtained DTI-derived brain structural connectomes and utilized vectorized structural connectivity as the node features (Figure 1B). We calculated the similarity between brain structural connectomes as the weight $W\left(i,j\right)$ of edge E between subjects *i* and *j* (Figure 1C). Additional details are elaborated in the cohort graph construction section.

### 2.2. Data Acquisition and Processing

#### 2.2.1. Subjects and MRI Data Acquisition

The Cincinnati Children’s Hospital Institutional Review Board approved this study. A parent or guardian of each infant gave written informed consent before enrollment. A total of 264 very preterm infants from five level-III Greater Cincinnati area neonatal intensive care units were enrolled at the time this work commenced. Cohort exclusion criteria included: (1) subjects with cyanotic heart disease or chromosomal or congenital anomalies affecting the central nervous system; (2) subjects who were hospitalized and mechanically ventilated on more than 50% supplemental oxygen at 45 weeks postmenstrual age (PMA). All experimental protocols involving human subjects were performed in accordance with the Declaration of Helsinki.

All study infants were imaged during unsedated sleep between 40 and 44 weeks postmenstrual age on a 3T Philips Ingenia scanner (Eindhoven, The Netherlands) with a 32-channel receiver head coil. All MRI scans were performed at Cincinnati Children’s Hospital. A skilled neonatal nurse and neonatologist were both present for any scans requiring positive pressure airway support. The MRI data acquisition parameters were as follows: B800 DTI: echo time, 88 ms; repetition time, 6972 ms; flip angle, 90°; field of view, 160 × 160 mm^2^; 80 × 79 matrix; 2 mm contiguous slices; scan time, 5:58 min. Thirty-six directions of diffusion gradients were applied with a b-value of 800 s/mm^2^; low b-value = 0 (4 b0 images were acquired with posterior–anterior phase encoding, and 1 b0 image was acquired with anterior–posterior phase encoding); axial T2-weighted MRI: echo time, 166 ms; repetition time, 18,567 ms; flip angle, 90°; voxel dimensions, 1.0 × 1.0 × 1.0 mm^3^; scan time, 3:43 min.

#### 2.2.2. MRI Data Preprocessing and Feature Extraction

We excluded 40 subjects due to severe brain injuries or large motion artifacts. The final cohort in this work contained 224 very preterm infants. For each subject, the DTI data were preprocessed using FMRIB’s Diffusion Toolbox (in the FMRIB Software Library, FSL, FMRIB Analysis Group, 6.0.1, Oxford, UK). Diffusion tensor reconstruction and brain fiber tracking used Diffusion Toolkit (0.6.4, Massachusetts General Hospital, Boston, MA, USA)/TrackVis (0.6.1, Massachusetts General Hospital, Boston, MA, USA) [28]. Head motion and eddy current artifacts were mitigated by aligning all diffusion images to their B0 image via an affine transformation. The fiber tracking was performed employing a deterministic tracking algorithm and used an angular threshold of 35 degree with a fiber length threshold of 5 mm [29]. The whole-brain structural connectome was constructed based on 90 regions of interest (ROIs) defined form a neonatal automated anatomical labeling (AAL) atlas [30]. The structural connections between each pair of ROIs were calculated as the mean fractional anisotropy of each voxel intersecting the tract and then averaged over all tracts between the two ROIs, resulting in a 90 × 90 symmetric adjacency matrix. This was performed using the UCLA Multimodal Connectivity Package (1.1, UCLA, Los Angeles, CA, USA) [31].

#### 2.2.3. Motor Abnormalities Evaluation at 2 Years Corrected Age

Motor abnormalities evaluation was conducted for our subjects at 2 years corrected age as the gold-standard reference. One hundred and nineteen preterm infants had received the standardized Bayley Scales of Infant and Toddler Development III (Bayley-III) test at 2 years corrected age, and the remaining 105 infants had not yet reached this age at study commencement [32]. The Bayley-III Motor function composite (of fine and gross motor) sub-score was utilized to evaluate the risk of motor abnormalities for individual very preterm infants. The Bayley-III motor scores were normalized to a scale of 40–160, with a mean of 100 and a standard deviation of 15. We chose one standard deviation below the mean (i.e., 85) as the cutoff value to dichotomize the cohort into a high-risk group (motor score ≤ 85; 37 subjects) and a low-risk group (motor score > 85; 82 subjects) in terms of developing moderate/severe motor abnormalities.

### 2.3. Semi-Supervised Graph Convolutional Networks

#### 2.3.1. Construction of the Initial Cohort Graph

Graph construction is an important foundation for our semi-supervised learning framework. In this work, we constructed an *initial cohort graph* for the semi-supervised GCN to learn the features of individual subjects and capture their relationships. Specifically, we represented the very preterm infant cohort using a weighted graph $G\left(𝓋,E,\;W\right)$, where each node 𝓋 of the graph represented a very preterm infant, and the weighted edge E represented the relationship between two infants associated with a weight W. This cohort graph contained all very preterm infants with or without 2-year motor function follow-up assessment tests. Each node 𝓋 was associated with a set of features. In our setting, we extracted and vectorized the unique connectivity weights of individual brain structural connectomes as a vector of 4005 features. Furthermore, we applied the natural exponential of Euclidean distance between node features (i.e., vectorized brain connectomes) as the similarity/relationship between very preterm infants. As in a prior study [20], the edge weight between nodes *i* and *j* can be calculated by

$$W\left(i,j\right)=e^{-\frac{d\left(SC\left(i\right),\;SC\left(j\right)\right)}{2\sigma^{2}}}$$

where d is the Euclidean distance, $SC\left(i\right)$ and $SC\left(j\right)$ are the brain structural connectome features of subjects i and j, and σ is a coefficient that determines the edge weight distribution.

**Figure 2:**
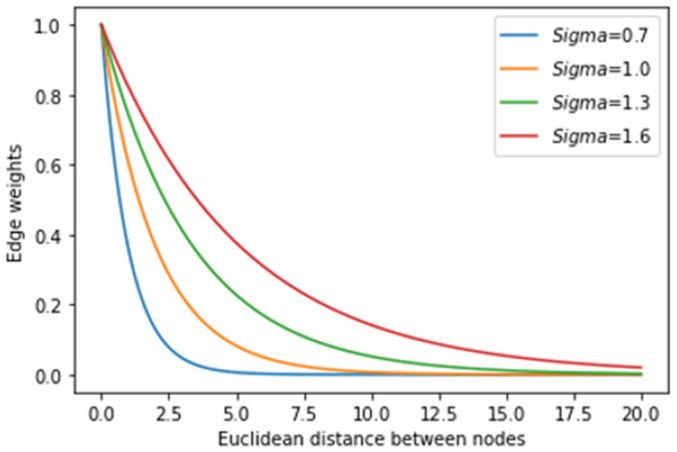
Edge weights between two nodes are dependent on the Euclidean distance between the brain connectomes of two nodes. Various coefficients σ (i.e., sigma values) determine the edge weight distribution.

The edge weight W is a number between 0 and 1. Figure 2 shows how edge weights between two nodes change according to the Euclidean distance between the brain connectomes of two nodes. When the Euclidean distance between node features is zero, that is, when two nodes have the same features, the edge weight is 1. When the Euclidean distance between node features increases, the edge weight decreases. When the Euclidean distance between node features is infinite, the edge weight becomes zero. The rationale is that subjects with the same labels (high-risk or low-risk motor impairments) tend to have more similar brain structural connectomes than subjects with different labels. The coefficient σ can be optimized to control the edge weight distribution for specific applications. In this work, we optimized graph construction using various coefficients σ = [0.7, 1.0, 1.3, 1.6, 1.9, 2.2] based on prediction performance. As such, we defined the *initial cohort graph* for our very preterm infants with explicit node features and edge weights.

#### 2.3.2. Architecture of the Graph Convolutional Network

In this section, we elaborate the architecture of the semi-supervised GCN model for the early motor impairment risk prediction task. Given the constructed *initial cohort graph* $G\left(𝓋,E,\;W\right)$, the adjacency matrix of the graph is represented as $A\in {\mathbb{R}}^{N\times N}$, and the node feature matrix is represented as $X\in {\mathbb{R}}^{N\times M}$, where N is the number of subjects/nodes and M is the number of features (i.e., 4005 brain structural connectivity weights). The graph convolutional layer encodes the nodes with a forward propagation rule:

$$H^{l+1}={\tilde{D}}^{-\frac{1}{2}}\tilde{A}{\tilde{D}}^{-\frac{1}{2}}H^{l}W^{l}$$

where $\tilde{A}=A+I$ is the adjacency matrix with added self-connections and I is the identity matrix. $W^{l}\;\in {\mathbb{R}}^{M\times F}$ is the weight matrix of graph convolutional filters in the layer l. ${\tilde{D}}^{-\frac{1}{2}}\tilde{A}{\tilde{D}}^{-\frac{1}{2}}$ is the approximation of the normalized graph Laplacian of the weighted graph $G\left(𝓋,E,\;W\right)$, developed by [22] using a truncated expansion in terms of Chebyshev polynomials. $H^{l+1}$ is the node feature embedding matrix of layer l+1, aggregating the node feature embedding $H^{l}$ from layer l using the graph adjacency matrix A, and $H^{0}=X$.

**Figure 3:**
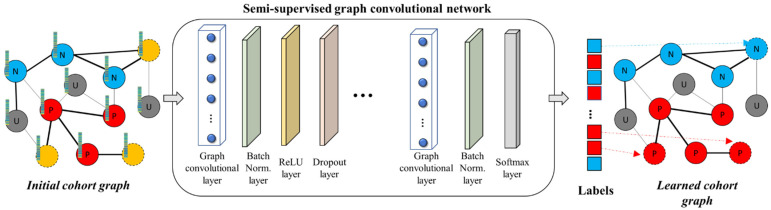
Architecture of the semi-supervised graph convolutional network (GCN) model. The model learns the whole cohort graph and outputs labels for individual nodes/subjects. Our GCN model consists of L hidden graph learning blocks and a final output block with a SoftMax layer for classifying graph nodes. Labeled samples are marked blue and red, Unlabeled samples are marked gray. New samples are marked yellow. P: positive, N: negative, U: unlabeled.

We demonstrate the architecture of the developed semi-supervised GCN model in Figure 3. Our GCN model consists of a series of L graph learning blocks. Each of the first L−1 graph learning blocks is made of a graph convolutional layer with k graph convolutional filters, a batch normalization layer, a rectified linear unit (ReLU) activation layer, and a dropout layer. At the end, we append a block with a graph convolutional layer, a batch normalization layer, and a SoftMax layer as the output of the GCN model for label classification. The number of hidden graph learning blocks is selected from [1, 2, 3, 4]. The number of graph convolutional filters is selected from [512, 256, 128, 64, 32, 16], according to a prior DTI study [33].

#### 2.3.3. Model Training

The proposed semi-supervised GCN model can be trained using the binary cross-entropy loss function:

$$L=-\frac{1}{N}\overset{N}{\underset{i=1}{\sum }}y_{i}\log\left(p\left(y_{i}|SC\left(i\right)\right)\right)+\left(1-y_{i}\right)\log\left(1-p\left(y_{i}|SC\left(i\right)\right)\right)$$

where $p\left(y_{i}|SC\left(i\right)\right)$ is the probability of the *i*th subject’s structural connectome $SC\left(i\right)$ being classified as the label $y_{i}$. However, since only a small portion of the cohort were at high risk for motor abnormalities, the sample ratio between the high-risk and low-risk motor impairment groups was highly imbalanced—a unique challenge that was not encountered in prior graph-based studies [24]. To mitigate the impact of such an imbalanced dataset issue, we considered a weighted binary cross-entropy loss function:

$$L=-\frac{1}{N}\overset{N}{\underset{i=1}{\sum }}\beta_{i}y_{i}\log\left(p\left(y_{i}|SC\left(i\right)\right)\right)+\beta_{i}\left(1-y_{i}\right)\log\left(1-p\left(y_{i}|SC\left(i\right)\right)\right)$$

where $p\left(y_{i}|SC\left(i\right)\right)$ is the probability of the *i*th subject’s structural connectome $SC\left(i\right)$ being classified as the label $y_{i}$. The class weight $\beta_{i}=\frac{N}{C_{i}}\;$ is computed using the inverse frequency method, where the variable $C_{i}$ is the total number of samples for the class group of subject i.

To train the GCN model, we applied the Adam algorithm [34] to optimize the weights of the model with an initial learning rate of 0.01, the first and second decay rates being 0.9 and 0.999. We set a maximal epoch of 2000. During model training, the whole *initial cohort graph* (adjacency matrix A) and their feature vectors (feature matrix X) were inputted into the model. Only a training subset of the graph nodes was labeled. The remaining graph nodes (either unlabeled subjects or to-be-classified subjects in the testing set) can be seen by the GCN model without any label information during training. The features of these unlabeled nodes/subjects would impact the graph convolutions of labeled subjects when the GCN model performed the forward propagation. This makes the GCN model learn both labeled subjects (features and labels) and unlabeled subjects (features only) in a semi-supervised manner.

#### 2.3.4. Model Evaluation

We evaluated the classification performance of the proposed semi-supervised GCN model through 5-fold cross-validation. The labeled subjects were partitioned into 5 portions. While one portion of labeled subjects was used for testing, the other 4 portions were used for model training. The unlabeled subjects (i.e., very preterm infants without 2-year follow-up tests) were included in the training procedure to aid the GCN model training and were not included in the testing procedure. This process was repeated 50 times to evaluate model performance variations.

We also compared our semi-supervised GCN model with several peer machine learning and deep learning models that have been applied in brain structural connectome studies [35,36], including a logistic regression model, a ridge classifier model, linear and nonlinear support vector machine (SVM) models, and deep neural networks. To handle imbalanced datasets, weighted variations of the aforementioned models were utilized if applicable. We trained weighted SVM models and also applied weighted cross-entropy loss functions to train the deep neural networks. After all of the models were trained, we evaluated the model performance by calculating the means and standard deviations (SDs) of five performance metrics, including accuracy, balanced accuracy, sensitivity, specificity, and area under the receiver operating characteristic curve (AUC), for binary risk classification of motor abnormalities.

## 3. Results

### 3.1. Subjects

We had a final cohort of 224 very preterm infants in this study after data exclusion ( Section 2). Among them, 119 subjects had received the standardized Bayley III test at 2 years corrected age (labeled subjects), while the other 105 subjects (unlabeled subjects) had not reached 2 years of age when this study commenced. A cutoff value of 85 (i.e., one standard deviation below the mean Bayley III motor score) was utilized to dichotomize the labeled subjects into groups at high risk (≤85; 37 subjects) and at low risk (>85; 82 subjects) of developing moderate/severe motor abnormalities. Detailed demographics of the study cohort are listed in Table 1.

### 3.2. Semi-Supervised GCN Model Optimization

**Figure 4:**
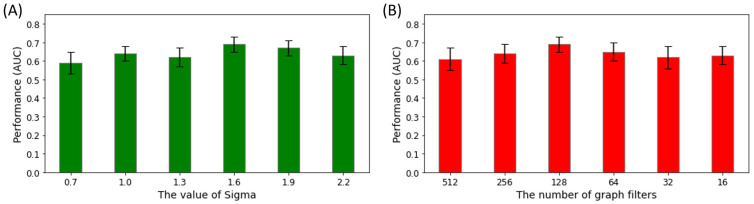
The model performance of the graph convolutional network model using (**A**) various edge weight coefficients for *initial cohort graph* construction and (**B**) different numbers of graph convolutional filters in individual layers.

We first conducted optimization experiments to explore the best hyperparameters, including the weight coefficient σ, the number of graph filters, and the number of graph layers. Figure 4A demonstrates the model classification performance in terms of AUCs when the *initial cohort graph* was constructed using various weight coefficients σ. Given a GCN model with three layers and 128 graph filters in each layer, the best prediction AUC was achieved when the *initial cohort graph* was constructed using coefficient σ=1.6. This is likely because the *initial cohort graph* (σ=1.6) was optimally constructed to model pairwise relationships between subjects. Similarly, Figure 4B shows the model’s AUCs obtained by three-layer GCN models with different numbers of graph filters in individual layers. For the *initial cohort graph* (σ=1.6), the AUC of our GCN model increased as the number of graph filters decreased. It reached peak performance (0.69 ± 0.04) when the number of filters was 128 and then decreased along with the decrease in the number of filters.

**Figure 5:**
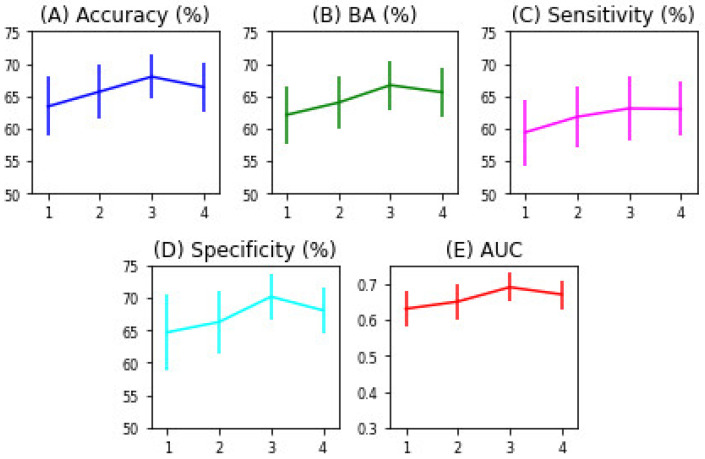
The model performance of the graph convolutional network model using different numbers of graph convolutional layers. The x-axis represents the number of graph convolutional layers, while the y-axis indicates the model performance. (**A**) Accuracy. (**B**) Balanced accuracy (BA). (**C**) Sensitivity. (**D**) Specificity. (**E**) Area under the receiver operating characteristic curve (AUC).

Next, we continued to optimize the GCN model by searching for the optimal number of graph convolutional layers in the GCN model. We trained the GCN models with layers $l=\left[1,2,3,4\right]$, and prediction performance with respect to motor impairment risk was evaluated on testing subjects using the *learned cohort graph*. As shown in Figure 5, the model performance increased as we increased the number of graph convolutional layers. The model performance started to drop when the number of layers in the GCN model reached four layers. Thus, we obtained an optimal architecture for our GCN model, which consists of three graph learning blocks. The first and second blocks are made of a graph convolutional layer with 128 graph convolutional filters, a batch normalization layer, a ReLU activation layer, and a dropout layer. The third block contains a graph convolutional layer with 128 graph filters, a batch normalization layer, and a SoftMax layer as the output of the model. The optimal accuracy (68.0% ± 3.4%), balanced accuracy (66.7% ± 3.8%), and AUC (0.69 ± 0.04) were achieved by this optimized GCN model.

### 3.3. Performance Comparison with Other Models

Next, we compared the proposed GCN model with several peer models (Section 2). Table 2 shows the model performance comparison for our early motor impairment prediction task. To investigate the effects of unlabeled data, we also trained the GCN model with and without unlabeled subjects. With the help of unlabeled data, the semi-supervised GCN model was able to identify subjects at high risk of developing moderate/severe motor impairment with a mean accuracy of 68.0% and a mean AUC of 0.69, outperforming the other peer models.

Among peer supervised learning models, the nonlinear SVM model achieved the best performance with a mean accuracy of 66.2% and an AUC of 0.65. Although the ridge classifier reached a specificity of 71.9%, this superior performance was only due to its low capability to handle the imbalanced dataset, and its sensitivity was undesirably low at 50.5%. Our semi-supervised GCN model without unlabeled data achieved a significantly higher AUC (*p* = 0.029) than the nonlinear SVM model, although the accuracy difference was not significant (*p* = 0.476). This validated our hypothesis that the semi-supervised GCN model is able to improve prediction performance over peer supervised machine learning and deep learning models.

Our GCN model with additional unlabeled data achieved improved prediction performance over the GCN model without using any unlabeled data. By taking advantage of 105 unlabeled data, the model had significantly better prediction performance in terms of accuracy (68.0% vs. 66.4%, *p* = 0.016) and AUCs (0.69 vs. 0.67, *p* = 0.029). Our semi-supervised GCN model also achieved better performance than a nonlinear SVM model, with an increase of 1.8% in accuracy (*p* = 0.014) and 0.04 in AUC (*p* < 0.001). This validated our hypothesis that the integration of labeled and unlabeled data is able to boost the performance of GCN models that used labeled data alone.

### 3.4. Impact of Weighted Loss Functions

**Figure 6:**
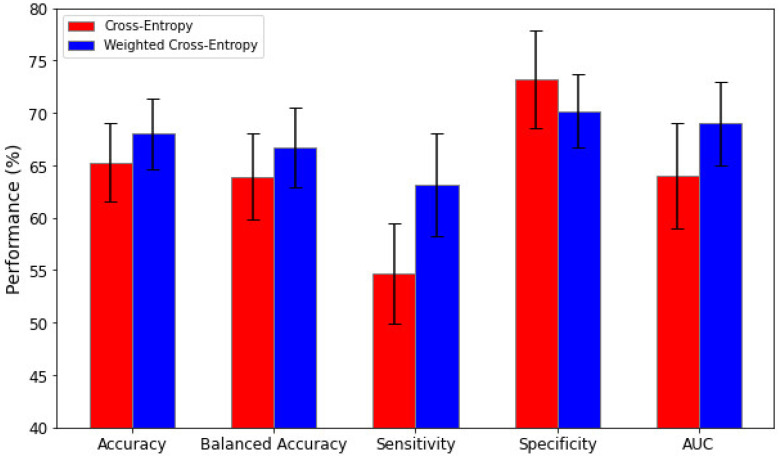
The model performance comparison of the graph convolutional network model using cross-entropy and weighted cross-entropy loss functions.

To investigate the impact of weighted loss functions, we compared the performance of our semi-supervised GCN model using either typical cross-entropy or weighted cross-entropy loss functions. Figure 6 displayed the bar plots of five performance metrics from the GCN models with two different loss functions. The model using the typical cross-entropy loss function achieved a low sensitivity of 54.7 ± 4.8% and a high specificity of 73.2 ± 4.7%, indicating that this model tends to place more subjects in the low-risk group than in the high-risk group. In contrast, the GCN model using the weighted loss function reached a better equilibrium with an improved sensitivity of 63.1 ± 4.9% and a slightly lower specificity of 70.2 ± 3.5%. Such a better equilibrium was also reflected in the higher accuracy, balanced accuracy, and AUC of the semi-supervised GCN model trained with the weighted cross-entropy loss function.

## 4. Discussion

Early identification of very preterm infants who may develop moderate/severe motor impairment is critical to design early personalized clinical intervention for improving their quality of life. In this work, we developed a semi-supervised GCN model for the early prediction of motor abnormalities at 2 years of age in very preterm infants using both labeled and unlabeled neuroimaging data. Our GCN model achieved a mean accuracy of 68.0% and a mean AUC of 0.69 on a partially labeled cohort of 224 very preterm infants and significantly outperformed multiple prior machine learning and deep learning models.

### 4.1. Comparison of the GCN Model with Other Models

Several prior studies have used DTI-derived features to predict motor abnormalities of preterm infants using supervised learning models. Chau et al. [14] documented that multiple brain regional fractional anisotropy values measured by DTI were associated with adverse motor development of very preterm infants. Brown et al. [15] calculated topological features (e.g., node degrees, clustering coefficients, and global efficiencies) of brain structural connectomes derived from DTI data for individual very preterm infants, then applied SVM models to discriminate between normal and abnormally low scores of motor development. In another study, Kawahara et al. [16] proposed a deep learning framework with customized convolutional filters to predict motor developmental scores by using brain structural connectomes derived from DTI data for preterm infants. More recently, we developed a deep convolutional neural network (CNN)-based multi-modal learning model to integrate DTI data together with other data modalities to predict motor abnormalities in very preterm infants [17]. However, these prior supervised learning models [14,15,16,17] in the context of neurodevelopmental outcome prediction did not consider modeling pairwise interactions between subjects and rather relied on subject-specific imaging feature vectors, limiting the full potential of the discriminative ability of neuroimaging data.

Graph-based approaches have been successfully applied in disease diagnosis applications [20,21]. Zhao et al. [20] constructed a compact population graph by representing AD and normal control subjects as nodes and deriving edge weights using a local reconstruction method. Then, they conducted a node classification in the constructed population graph to classify AD patients. Later, Tong et al. [21] developed a nonlinear graph fusion approach to merge multi-modality data into a unified graph for AD classification. However, these earlier studies mainly focused on pairwise relationships between subjects and did not consider features of individual subjects. Compared to other models, the GCN model is able to simultaneously learn neuroimaging features of subjects and consider the pairwise similarity between neuroimaging features during the model training procedure. Thus, GCN models have achieved state-of-the-art classification results in multiple applications for disease diagnosis. For example, Parisot et al. [24] applied GCN models in the diagnosis of ASD and AD. In this study, a vector of neuroimaging features was associated with nodes that represented subjects, and non-imaging features were used to model pairwise relationships between subjects as edge weights. Then, GCN models were applied to learn constructed population graphs and classify individual nodes into normal control or disease groups. Theoretically, GCNs could be considered as generalizations of the classic CNN model. Different from CNN models that operate on regular grid-like data/images using a fixed receptive field, GCN models are able to capture both local and global information in an irregular graph. Our GCN model outperformed other models in the early prediction of motor impairment in preterm infants. This is consistent with prior studies [20,21] using GCN models in other disease diagnosis applications.

### 4.2. Architecture Optimization of the GCN

In our work, we optimized our model by examining multiple hyperparameters, including the edge weight coefficient, the number of graph filters, and the number of graph layers. Our results showed that prediction performance reached a peak when our model had three graph convolutional layers. The performance started to decrease once we added more layers to the GCN model. The final GCN model contains three graph convolutional layers, each of which has 128 graph filters. The optimal depth of our GCN model depends on several factors, such as the complexity of the motor impairment prediction task. GCN models with one or two layers are easy to train with a smaller dataset due to fewer trainable weights. However, they may not have enough capacity to learn in such a complex prediction task. On the other hand, GCN models with more layers may have dramatically increased numbers of trainable weights of graph filters. Without a large dataset, simply adding more layers to the GCN model may cause an overfitting issue in our early-prediction task. Therefore, our GCN model with three layers reached the right balance between GCN model complexity and our dataset size.

### 4.3. Semi-Supervised Learning Using Unlabeled Datasets

Furthermore, most prior early-prediction studies mainly relied on labeled data to train supervised learning models. Typically, a large number of labeled training data are essential to develop robust models with desirable generalizability. Unfortunately, labeled training data are commonly very limited in most existing motor abnormalities prediction studies, since it is very challenging to collect large-scale neuroimaging datasets, especially ones with outcome data [37,38]. Acquiring later outcome data requires long-term follow-up of subjects to obtain clinical diagnoses. In this sense, a semi-supervised learning strategy that combines additional unlabeled data with labeled data during training may facilitate the development of prediction models. It is relatively easy to acquire unlabeled data, since clinical diagnoses or long-term follow-ups are not necessary. This means that it is feasible to significantly enrich a dataset for model development. Our results (Table 2) suggest that a semi-supervised GCN model is capable of leveraging additional unlabeled data to further improve the prediction of motor abnormalities.

### 4.4. Weighted Loss Functions for Imbalanced Datasets

The imbalanced dataset issue is a unique challenge in early-prediction studies of neurodevelopment in very preterm infants [17,35,39]. There are often significantly fewer subjects who develop moderate–severe motor abnormalities than who do not. Although this fact is desirable for infants, it creates a challenge for those developing machine learning models. When there are significantly more subjects in one group (referred to as the majority group), a model tends to assign undesirably more subjects to this majority group (i.e., low-risk motor impairment in this study) than to the minority group that contains fewer subjects (i.e., high-risk motor impairment). To reduce the impact of such imbalanced dataset issues, we applied a weighted cross-entropy loss function to train our semi-supervised GCN model. The results showed that the weighted loss function was effective to handle this problem and prevent the model being biased toward the majority group.

### 4.5. Limitations

There are certain limitations to this current study. First, the main limitation of the current work is that the semi-supervised GCN model represents a type of transductive learning, which requires training data to be available when the trained model infers an unseen new sample. This characteristic may limit the model’s applications on certain low-memory devices, such as mobile devices. Second, the graph construction strategy is key for a GCN model to perform a node classification task. An inappropriately constructed graph may negatively impact the learning procedures of semi-supervised GCN models. To generalize the method and achieve optimal performance in other applications, tailored adjustments or modifications to the proposed graph construction may be necessary. Finally, although we demonstrated that our semi-supervised GCN model is able to mitigate the impact of small datasets, it is possible to further improve the model’s performance by using additional labeled DTI data.

## 5. Conclusions

In this study, we strived to develop a semi-supervised GCN model for the early prediction of motor impairment at 2 years corrected age in preterm infants using both labeled and unlabeled DTI-derived brain structural connectomes. We demonstrated that the prediction performance of our proposed semi-supervised GCN model exceeded that of several prior supervised learning models. This semi-supervised GCN model can be generalized to aid early prediction of various neurodevelopmental deficits in very preterm infants.

## Figures and Tables

**Table 1:** Demographics of the subjects in the high-risk group, the low-risk group, and the unlabeled group.

	High-Risk Group	Low-Risk Group	Unlabeled Group
Number of subjects	N = 37	N = 82	N = 105
Gestational age at birth (weeks)	28.9 (2.6)	29.3 (2.4)	29.5 (2.5)
Postmenstrual age at the scan (weeks)	42.7 (1.1)	42.0 (1.3)	43.0 (1.2)
Female, N (percentage)	11 (29.7%)	43 (52.4%)	45 (42.8%)
Birth weight (gram)	1244.7 (462.8)	1276.0 (424.4)	1353.7 (429.1)

Continuous variables are listed as mean (standard deviation).

**Table 2:** Performance comparison for the prediction of very preterm infants at high risk vs. low risk of developing moderate/severe motor abnormalities at 2 years corrected age.

Models	Accuracy (%)	BA (%)	Sensitivity (%)	Specificity (%)	AUC
Logistic Regression	60.1 ± 4.0	58.7 ± 3.9	51.6 ± 4.4	65.7 ± 4.0	0.59 ± 0.03
Ridge Classifier	63.3 ± 3.7	61.2 ± 3.8	50.5 ± 4.2	71.9 ± 3.5	0.62 ± 0.04
SVM (linear kernel)	65.7 ± 3.4	64.5 ± 3.7	59.8 ± 5.1	69.1 ± 3.2	0.64 ± 0.03
SVM (rbf kernel)	66.2 ± 3.8	64.9 ± 4.0	61.5 ± 4.3	68.2 ± 4.1	0.65 ± 0.04
Deep neural network	65.9 ± 3.2	64.6 ± 4.4	60.2 ± 4.9	68.9 ± 4.3	0.64 ± 0.05
GCN (ours)	66.4 ± 3.3	65.4 ± 3.4	61.7 ± 4.7	69.0 ± 3.9	0.67 ± 0.05
GCN w/unlabeled (ours)	68.0 ± 3.4	66.7 ± 3.8	63.1 ± 4.9	70.2 ± 3.5	0.69 ± 0.04

Data are presented as mean ± standard deviation. BA—Balanced Accuracy; AUC—area under the receive operating characteristic curve; SVM—Support vector machine; rbf—radial basis function; GCN—graph convolutional network. GCN w/unlabeled—GCN trained with labeled and unlabeled data. All other models were trained with labeled data in a supervised manner.

## Data Availability

Requests to access the datasets used in this study should be directed to the corresponding author with a formal data sharing agreement and approval from the requesting researcher’s local ethics committee.
